# Activity in primate visual cortex is minimally driven by spontaneous movements

**DOI:** 10.1038/s41593-023-01459-5

**Published:** 2023-10-12

**Authors:** Bharath Chandra Talluri, Incheol Kang, Adam Lazere, Katrina R. Quinn, Nicholas Kaliss, Jacob L. Yates, Daniel A. Butts, Hendrikje Nienborg

**Affiliations:** 1 Laboratory of Sensorimotor Research, National Eye Institute, National Institutes of Health, Bethesda, MD USA; 2 Center for Integrative Neuroscience, University of Tübingen, Tübingen, Germany; 3 Herbert Wertheim School of Optometry & Vision Science, University of California, Berkeley, Berkeley, CA USA; 4 Department of Biology and Program in Neuroscience and Cognitive Science, University of Maryland, College Park, MD USA

**Keywords:** Sensory processing, Striate cortex, Extrastriate cortex

## Abstract

Organisms process sensory information in the context of their own moving bodies, an idea referred to as embodiment. This idea is important for developmental neuroscience, robotics and systems neuroscience. The mechanisms supporting embodiment are unknown, but a manifestation could be the observation in mice of brain-wide neuromodulation, including in the primary visual cortex, driven by task-irrelevant spontaneous body movements. We tested this hypothesis in macaque monkeys (*Macaca mulatta*), a primate model for human vision, by simultaneously recording visual cortex activity and facial and body movements. We also sought a direct comparison using an analogous approach to those used in mouse studies. Here we found that activity in the primate visual cortex (V1, V2 and V3/V3A) was associated with the animals’ own movements, but this modulation was largely explained by the impact of the movements on the retinal image, that is, by changes in visual input. These results indicate that visual cortex in primates is minimally driven by spontaneous movements and may reflect species-specific sensorimotor strategies.

## Abstract (web summary)

In primates, activity in the visual cortex is not driven by spontaneous body movements. These results confirm the functional specialization of primate visual processing, in contrast with findings in mice, and highlight the importance of cross-species comparisons.

## Main

Organisms process sensory information not in isolation but within the context of a moving body that is interacting with the environment. The importance of such embodiment is underscored in developmental neuroscience^1^ and in robotics and artificial intelligence^2^, from vacuum-cleaning robots to self-driving cars^3,4^ (https://www.youtube.com/watch?v=XY1VTLRIsNo). A longstanding question in systems neuroscience is the degree to which this embodiment influences sensory processing^5–7^. In mice, locomotion affects neural activity in primary visual cortex (V1)^8–19^ and spontaneous movements are associated with pronounced brain-wide activity including in V1 (refs. ^20–22^). The work in mice suggests that embodiment plays a crucial role in shaping processing in the visual cortex, although it is unclear whether similar phenomena are observed in other species^23–27^. The degree to which such movements influence responses in the primate visual cortex is of interest for several reasons. First, it could be a direct observation of embodiment that can be dissected into mechanisms and probed to understand its computational principles. Second, it addresses a fundamental question about the functional organization and degree of modularity of the primate cerebral cortex^24^. Third, it could have far-reaching implications for the interpretation of past neurophysiological studies of the primate visual system, in which the animals’ spontaneous body movements were not monitored.

In the present study, we asked whether the animal’s own body movements are associated with modulations of neural activity in visual cortex of macaque monkeys. We mirrored the experimental approaches used in studies in mice to facilitate the comparison between the data in mice and the data in primates: specifically, we used videography to monitor the animals’ movements^20,21^ and statistical modeling^21,22^ to relate the movements to neural spiking activity recorded in the visual cortex (V1, V2 and V3/V3A). Consistent with the results observed in mice, we found activity associated with the animals’ own spontaneous body movements. However, after accounting for the fact that some of these movements also changed the retinal input to the neurons in visual cortex, this movement-related activity largely disappeared. A model-free analysis confirmed that modulation by the animals’ own body movements was small. Moreover, using the model-free approach to compare the effect sizes, we found that a known modulator of visual cortical activity, the modulation by spatial attention, was about an order of magnitude larger than that by the animals’ own movements, and these two effects were not associated. We conclude that, in macaque early and mid-level visual cortex, activity is minimally driven by the animal’s own spontaneous body movements.

## Results

### Macaque monkeys move spontaneously while performing visual tasks

**Fig. 1:**
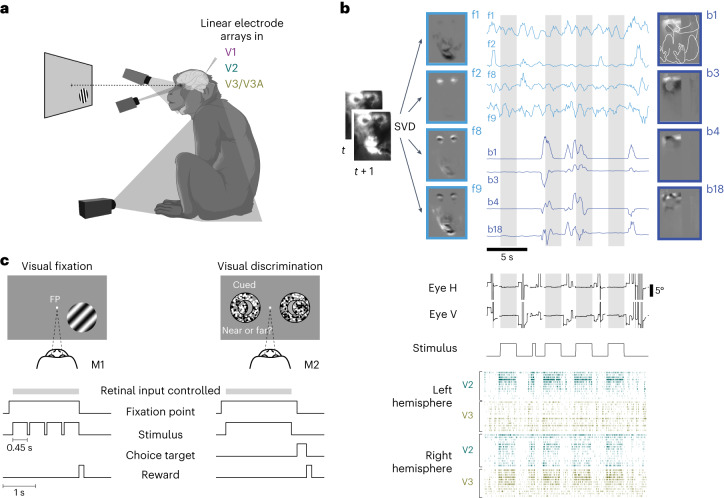
Monitoring spontaneous body movements during task performance in macaque monkeys. **a**, The set-up. The animals performed a visual task while extracellular activity in their visual cortex was recorded and the animals’ body, face and eye movements were monitored via video, with one camera directed at the body, one at the face and a video-based eye tracker. **b**, Movements recorded by video (example from M2) were decomposed (SVD), generating multiple components of face and body movements that map on to, for example, movements of the mouth (face component 1 (f1)), eye blinks (f2), combinations of face parts (f8 and f9) and combinations of hand, arm, leg and body movement (body components b1, b3, b4 and b18; outline of the monkey body shown in b1; grayscale shows normalized components; traces show normalized temporal profiles of the video projected on to the components); the middle panels show eye positions and stimulus ON/OFF periods. Dark-gray bands in the eye position traces indicate interrupted eye signals resulting from blinks or eccentric eye positions; light-gray shading marks epochs when the animals fixated and the retinal input was controlled. Bottom, sample spike rasters of simultaneously recorded units in the left and right hemispheres of V2 and V3/V3A. In each row spike times from one unit are shown as vertical ticks. **c**, Animal M1 performing a visual fixation task and animal M2 performing a visual discrimination task combined with block-wise manipulation of spatial attention. The retinal input was controlled during periods (gray bar) when the animals fixated on an FP at the center of the screen.

**Extended Data Fig. 1:**
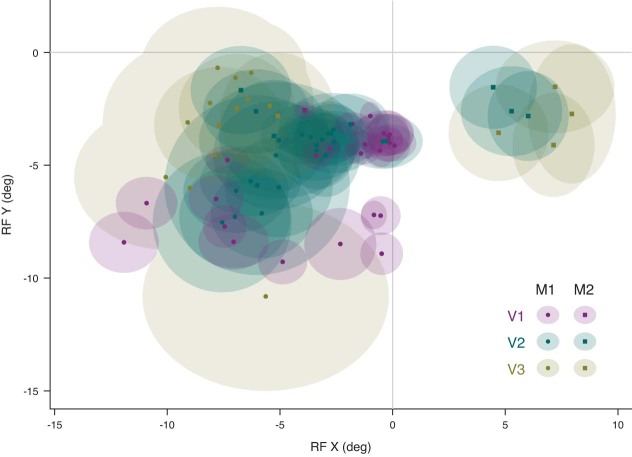
Receptive Field distribution. The average receptive field centers and widths (shaded ellipses) for each session and area are plotted for animal M1 (circles) and animal M2 (squares). Data for V1, V2, V3/V3A are shown in purple, green and brown, respectively. The median eccentricity of the receptive fields of the recorded units was 4.4° for V1 operculum (range: 2.9° ~ 7.3°), 10.5° for V1 calcarine sulcus (range: 8.5° ~ 14.4°), 6.5° (range: 3.4° ~ 10.8°) for V2, and 8.1° (range: 3.2° ~13.8°) for V3/V3A.

We used multichannel extracellular recordings targeting V1, V2 and V3/V3A (Extended Data Fig. 1) combined with video-based monitoring of the body and face in two alert macaque monkeys. The animals performed a visual fixation task or visual discrimination task (Fig. 1). They fixated a spot on the center of the display during stimulus presentation epochs, which allowed us to reconstruct the stimulus in retinal coordinates. Outside the stimulus presentation epochs, the animals freely moved their eyes. Similar to the mice in the previous studies^20–22^, the monkeys were head fixed, but otherwise free to move their arms, legs and bodies throughout and in between stimulus presentations while seated. Importantly, pronounced modulation by spontaneous movements was apparent even in a set-up in mice^21^ that did not allow the head-fixed, task-performing mice to locomote. This is directly comparable to the set-up in the macaques used in the present study. As the videography confirmed, the animals often fidgeted and moved spontaneously throughout the recording sessions (Fig. 1b and Supplementary Videos 1–3). We directed one camera at each animal’s face and one at the animal’s body. The videos thus captured movements of the hands, feet, limbs and trunk, as well as the movements of the face such as licking, eye movements and blinks. We defined all these movements as the animal’s spontaneous movements. (We note that our results were qualitatively similar when we defined licking movements as task related.) The only task-relevant instructed movements were animal M2’s saccades toward one of the two target locations to report choices. To identify the animals’ movement patterns from the videos we used singular value decomposition (SVD) analogous to previous work in mice^20^ (Fig. 1b). From these data, we could directly ask to what extent the animals’ own spontaneous face and body movements predict neural activity in the primate visual cortex.

### Spontaneous movements predict neural activity when the retinal input is uncontrolled

**Fig. 2:**
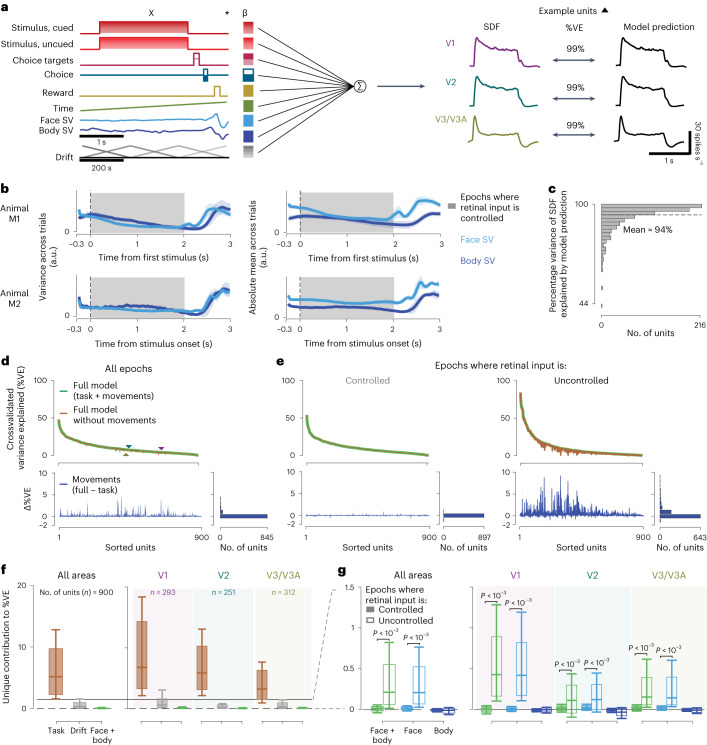
Body and face movement of the macaque monkey has minimal impact on neural activity in its visual cortex. **a**, Linear encoding model predicting neural firing in the visual cortex (the predictors, labels left, are for the task used in M2; for M1, see Extended Data Fig. 2). The drift predictor captures slow fluctuations across trials (note slower, 200-s timescale). The three traces show the peristimulus SDFs for sample units in V1, V2 and V3/V3A (left) and the model predictions (right). **b**, Mean variance (left) and absolute mean (right) of the top 30 face and body movement components across trials (M1, top; M2, bottom). Shaded error bars show s.d. across sessions and shaded area epochs during which the animals maintained visual fixation (controlled retinal input). a.u., arbitrary units. **c**, Histogram showing the distribution of %VE (median = 97%, *n* = 900 units from both animals) of the SDF by the model predictions across units. **d**, Top: variance explained across all time points by the model with (green) and without (brown) movement predictors for all units (%VE, mean = 9.8% and 9.67%, respectively). Triangles show the example units from **a** (differences in %VE in **a** and **d** largely result from spike count variability at these high time resolutions). Bottom: difference in variance explained by the two models, reflecting the %VE by movements. Units are ranked by their variance explained by the full model. **e**, Same as **d**, but separately for epochs when retinal input was controlled (left, shaded interval in **b**) and not controlled (right). **f**, Unique variance explained by different covariates toward the full model, for units across all areas (left; including 44 units for which the area could not be assigned) and separated by area (right). Box plots show the interquartile range, whiskers the range covering the 66th percentile of the data and solid lines inside the box plots the median across units. **g**, Unique variance explained by movement covariates toward the full model, separately for controlled and uncontrolled retinal input epochs, for units across all areas (left) and separated by area (right). Note the smaller *y* scale compared with **f**. Format as in **f**. *P* values were obtained using a two-sided permutation test, uncorrected for multiple comparisons. Source data

**Extended Data Fig. 2:**
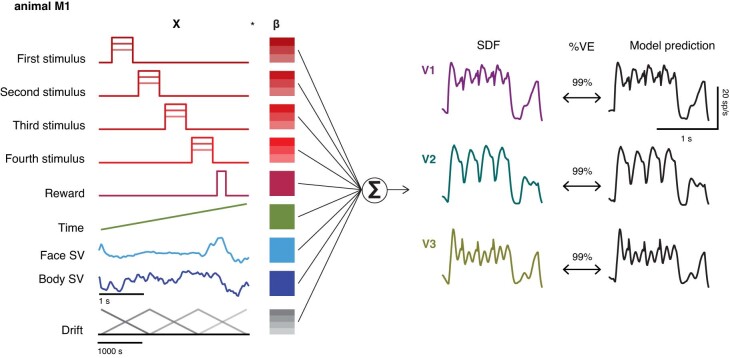
Schematic of the linear encoding model for animal M1. Linear encoding model predicts neural firing in visual cortex (the predictors, labels left, are for the task used in M1). The three traces show peristimulus spike-density function for example units in V1 (purple), V2 (green), and V3/V3A (brown; left) recorded in M1, the model predictions (right), and the variance explained by these predictions (center).

Following previous work in mice, we analyzed the data using a linear encoding model^21,22^ to predict the neural activity using a set of ‘predictors’ (Fig. 2a). The predictors included controlled variables in the experiment related to the task and the stimulus, uncontrolled but observable variables such as the temporal profiles of the movement components (Fig. 2a, labels on left and Extended Data Fig. 2) and temporally shifted versions of these predictors. The model successfully captured the stimulus-aligned response: the predicted firing rate at 16-ms resolution and the peristimulus spike density function (SDF) over all trials were closely matched (Fig. 2a, right, c). Such peristimulus, SDF-based validation, however, obscures the effects of spontaneous movements on both the model and the data, because the movements are not necessarily time locked to events in the trial. Indeed, although some movements were aligned with the trial events, there was substantial movement variability throughout the trial. The movement variability also included the stimulus presentation period, when the animals maintained visual fixation on a small dot in the center of the screen (Fig. 2b). Thus, to capture trial-to-trial variability, which included the potential role of the animal’s own body movements, we evaluated model performance on an individual trial level. To do so we computed the crossvalidated percentage variance explained (%VE; Methods), for each of the 900 units across both animals and all areas (Fig. 2d). Beyond allowing for a comparison with the results from mice, this linear encoding approach has several advantages (Discussion). For example, it can uniquely attribute modulatory effects to one of many covariates, even when they are partially correlated with each other^21^.

To address our central question of whether neural activity can be explained by the animal’s spontaneous movements, we compared two models: first, the full model, with all the predictors (Fig. 2d, full model, green), and second, a ‘task-only’ model (Fig. 2d, brown), which was the full model but with the contribution of the movement predictors removed (Methods). The difference in variance explained between these two models is a measure of the amount of variance that can be explained uniquely by knowing the animals’ own movements (Fig. 2d, bottom). This quantity, defined as ‘unique variance’^21^, is computed as the difference between the full model and a reduced model. It measures the modulation in spiking activity uniquely explained by a set of model predictors, in this case by the face/body movements. The results show that activity in the primate visual cortex was predictable from the animal’s own movements. Still, the size of this contribution in the macaques was smaller compared with that seen in mouse visual cortex^21,22^.

**Extended Data Fig. 3:**
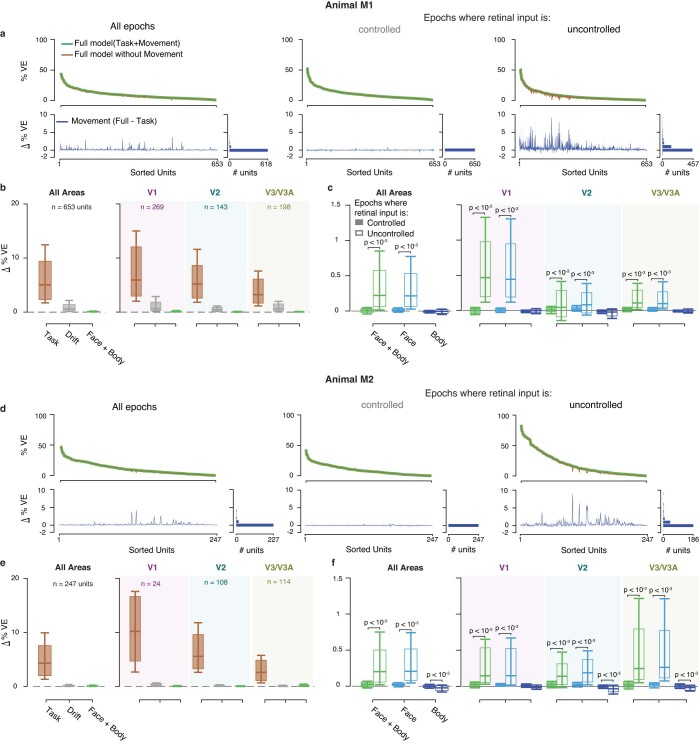
Linear encoding model fits, separately for animals M1(a-c) and M2 (d-f). **(a, d)** Top: Variance explained by the model with (green), and without (brown) movement covariates for all epochs (left), and separately for epochs when retinal input was controlled (middle), and not controlled (right) for all units. Bottom: Difference in variance explained by the two models. Units are sorted according to the variance explained by the full model. **(b, e)** Format as in Fig. 2f**(c, f)** Format as in Fig. 2g. For M1: during controlled retinal input epochs (**a**, middle), 3% of units (V1: 15/269, V2: 10/143, V3/V3A: 6/198), and during uncontrolled retinal input epochs, (**a**, right), 55% of units cross the threshold of ∆%VE > 0.1 (V1: 232/269, V2: 67/143, V3/V3A: 108/198); for M2: during controlled retinal input epochs (**d**, middle), 6% of units (V1: 0/24, V2: 6/108, V3/V3A: 7/114), and during uncontrolled retinal input epochs, (**d**, right), 66% of units cross the threshold (V1: 14/24, V2: 64/108, V3/V3A: 83/114). Box plots, inter-quartile range; whiskers, range covering 66 percentile of the data; solid lines inside the box plots, median across units. P-values obtained using two-sided permutation test, uncorrected for multiple comparisons. **b**, **c**, **e**, **f**: Data for V1, V2, V3/V3A are shaded in purple, green and brown, respectively.

To better understand how the monkeys’ own movements impacted neural activity in the visual cortex, we examined the unique variance associated with spontaneous movements during different epochs of the trial. First, when the retinal input was controlled because the animal maintained visual fixation (gray bar, Figs. 1c and 2b) and, second, when the retinal input was uncontrolled. In the first type of epoch, the retinal image (gray screen or the stimulus) is known and the corresponding predictors can contribute systematically to the model predictions. In the second type of epoch the retinal image is not known and thus could drive activity in a way that is predicted by movements causing these changes in retinal input. The animals moved spontaneously during both kinds of epochs (Fig. 2b). But the contribution of the model attributed to the movement almost completely disappeared when the retinal image could be inferred (Fig. 2e, left), compared with when the retinal input was uncontrolled (Fig. 2e, right). For each unit, we applied a threshold to determine whether the neural activity of the unit was associated with face or body movement (threshold, unique variance > 0.1 %VE). When the retinal input was controlled, 5% of units crossed the threshold (V1, 15 of 293; V2, 16 of 251; V3/V3A, 13 of 312), compared with 67% of units when the retinal input was uncontrolled (V1, 246 of 293; V2, 131 of 251; V3/V3A, 191 of 312). This result was robust for different thresholds of unique variance (Supplementary Table 1) and the increase in unique variance explained by movements when the retinal input was uncontrolled was significant (*P* < 0.001 for each area and combined across areas, by permutation test, Fig. 2g; similar in each animal individually, Extended Data Fig. 3). Given that neurons in these visual areas are driven by the visual stimulus, the higher unique variance explained by movements during the epochs when the visual stimulus was uncontrolled could readily result from changes in the visual stimulus because of these movements.

**Extended Data Fig. 4:**
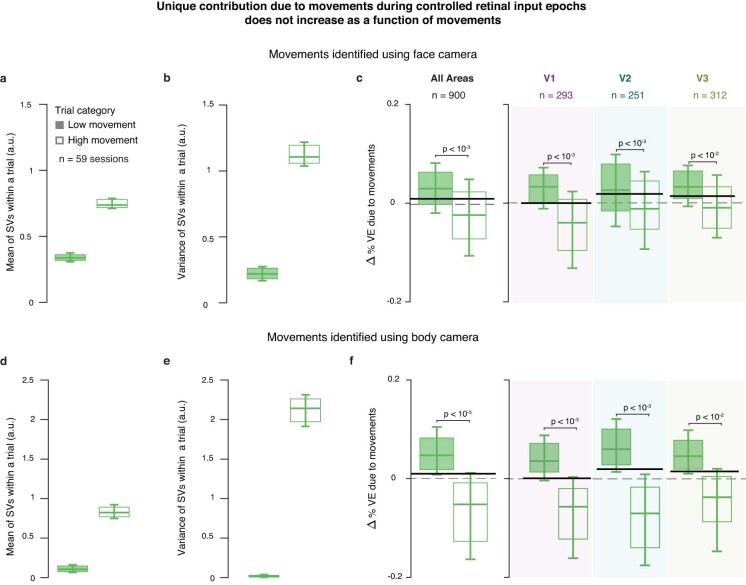
Unique contribution by movements decreases for trials with more movements suggesting an overfitting by movement regressors. We categorized trials into high and low movements trials based on the mean variance of the SVs within trials, averaged across all 30 SVs, during controlled retinal input epochs (top versus bottom third of trials). **(a-c):** Trials were categorized with SVs extracted from recordings of the face camera. **(a)** Mean of SVs within a trial separately for high and low movement trials. **(b)** Same as **(a)** but variance of SVs in a trial. **(c)** Unique variance explained by movements for units across all areas (left), and separately for each area (right). **(d-f)** Same as **(a-c)** but when trial categorization was done with SVs extracted from recordings of the body camera. The figure shows that movement regressors overfit trials with high movements, compared to trials with low movements. Box plots, inter-quartile range; whiskers, range covering 66 percentile of the data; solid lines inside the box plots, median across units. Solid black lines show the corresponding mean unique variance across all units for controlled retinal input epochs in all trials (for comparison, replicated from Fig. 2g). P-values obtained using two-sided permutation test, uncorrected for multiple comparisons. **c**, **f**: Data for V1, V2, V3/V3A are shaded in purple, green and brown, respectively.

In addition, on trials with more movements and more movement variability during the controlled retinal input epochs, the body and facial movements tended to explain even less neural variance (Extended Data Fig. 4). Our findings are therefore not the consequence of an overall paucity of movements by the macaques. Together, these results suggest that the larger unique contribution of the animal’s own movements when the retinal image was uncontrolled was the result of changes in the retinal image associated with these movements.

### Retinal input control reduces activity predicted by spontaneous movements

To validate this explanation, we compared the unique variance explained by movements inferred from the face view versus the body view. The explanation predicts that face movements, such as blinks or eye movements, are more likely than body movements to modulate neural activity. Consistent with this prediction, the increase in unique variance during epochs when the retinal input was uncontrolled was significant only for movements of the face (*P* < 0.001 for each area and combined across areas, permutation test, Fig. 2g). Moreover, removing the region of the eye from the face view reduced the increase in unique variance for retinal input-uncontrolled epochs (*P* < 0.001 for each area and combined across areas, permutation test). Conversely, the contribution by body movements was small throughout all epochs (Fig. 2g, unique variance due to body covariates, mean across epochs and units = −0.005 %VE, *P* = 0.07), mirroring previous findings in mice^21^.

**Extended Data Fig. 5:**
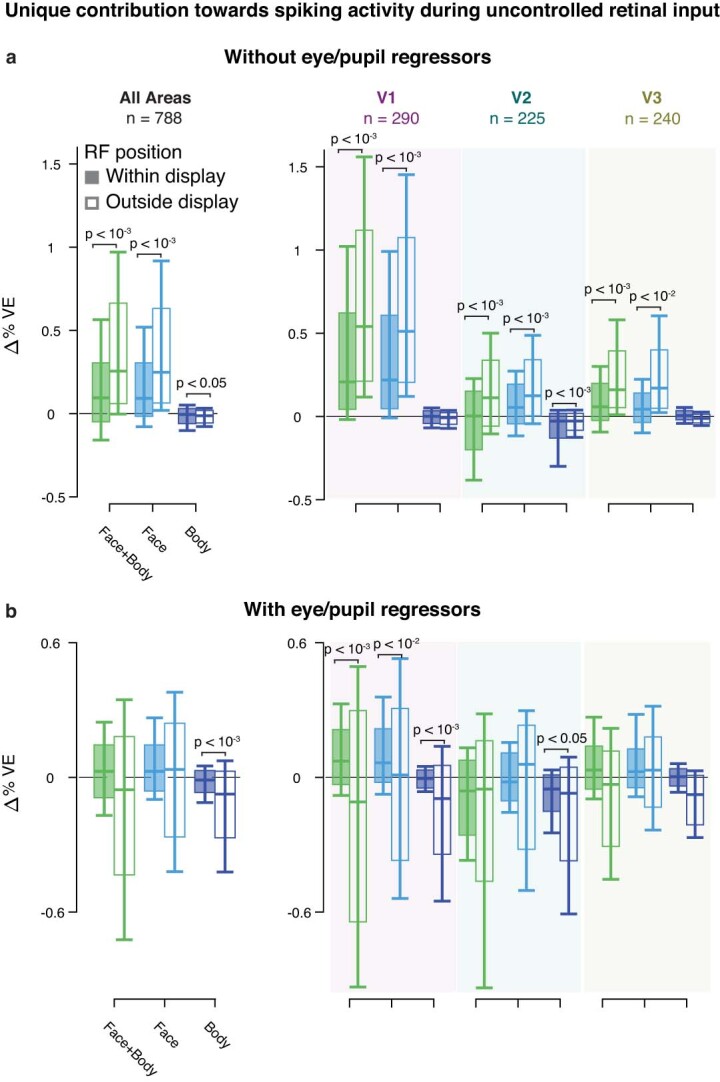
Movements have minimal effect on neural activity after controlling for eye movements in epochs when the animals do not maintain visual fixation. Unique variance explained by different covariates towards the full model during uncontrolled retinal input (open bars in Fig. 2g). **(a)** Model without eye & pupil regressors (see Extended Data Fig. 6). **(b)** Model including eye & pupil regressors. Format as in Fig. 2g. Unique variance was computed separately for time-points when the receptive field (RF) of the unit was on the monitor showing a gray screen (shaded bars; time-points when retinal input could be inferred), and when the receptive field was outside the boundaries of the monitor (open bars; time-points when retinal input could not be inferred). Location of the RF at different time points was reconstructed using gaze position obtained from recordings of the eye-tracker camera. The criterion for defining whether the RF was on the monitor was that the center of the RF + twice its width was within the monitor edges along the horizontal and vertical dimension. In addition to our general inclusion criteria (see Methods) we required that the unique variance take a finite value during both, inferred and uninferred time-points. For example, a unit whose receptive field was always inside the display during the session had no uninferred time-points, and hence had an undefined unique variance value for uninferred epochs. Box plots, inter-quartile range; whiskers, range covering 66 percentile of the data; solid lines inside the box plots, median across units. P-values obtained using two-sided permutation test, uncorrected for multiple comparisons. Data for V1, V2, V3/V3A are shaded in purple, green and brown, respectively.

**Extended Data Fig. 6:**
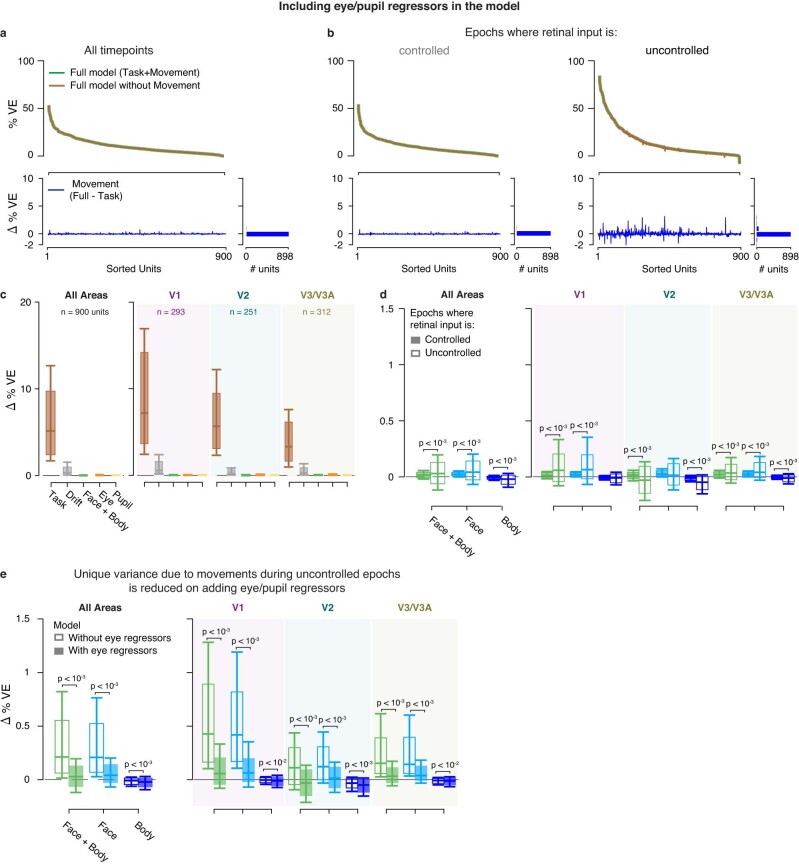
Linear encoding model fits, including eye and pupil covariates. Eye movements and pupil size were recorded using a separate camera (eye-tracker camera, see Methods). Horizontal and vertical eye movements, eye-speed, pupil diameter, and derivative of pupil diameter were used as additional regressors to model neural variability due to eye-movements. **(a-d)** Model fits for n = 900 units from both animals; format as in Fig. 2d–g. **(e)** Comparison of unique variance due to face and body movements during uncontrolled retinal input epochs between models with and without eye and pupil covariates. Adding the covariates substantially reduced the unique variance due to face movements during uncontrolled epochs, suggesting that the motion components extracted from the face camera also captured eye-movements that change the retinal input. Box plots, inter-quartile range; whiskers, range covering 66 percentile of the data; solid lines inside the box plots, median across units. P-values obtained using two-sided permutation test, uncorrected for multiple comparisons. **d**, **e**: Data for V1, V2, V3/V3A are shaded in purple, green and brown, respectively.

The data presented in the current study suggest that accounting for retinal input removes the variability of neural responses which was predictable from the monkey’s own movements. To further test this idea, we focused on all the time points during which the retinal input was uncontrolled. For all these time points the animals could move their eyes freely and we classified them into two subsets. The first subset is composed of times when the retinal input to the receptive fields of the recorded neurons could be inferred from the eye position. During these time points, the receptive fields were on a blank gray screen. The second subset is composed of times when the retinal input to the receptive fields could not be inferred. During these time points, the gaze of the animal could take receptive fields off the screen and they probably included visual structure from the room. If the absence of retinal image control can explain the apparent neural modulation by body/face components, then the neural modulation by the animal’s movements should be higher in the latter case when the retinal image is not known. This is exactly what we found (Extended Data Fig. 5a). This additional evidence suggests that the modulation by movement was largely attributable to eye movements because these eye movements changed the retinal input. To directly test this explanation, we added the eye position, eye velocity and pupil measurements obtained from separately performed eye tracking as regressors to the model (Extended Data Fig. 6). As predicted by this explanation, inclusion of the eye signals explicitly in the model substantially reduced the unique variance due to face movements during epochs with uncontrolled retinal input (Extended Data Figs. 5b and 6e). Together, these results support a relationship between spontaneous movements in primates and visual cortical activity because of their correlation with changes in the retinal input.

**Extended Data Fig. 7:**
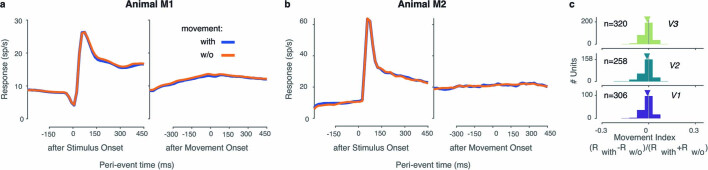
Model free analysis confirms the minimal modulation by movements. Trials were divided into two groups (‘with movement’ versus ‘without movement’ trials) depending on whether the animals moved on these trials. The movement was quantified as the average |pixel-difference| across consecutive video frames, and we used this metric to classify trials (see Methods). The average stimulus-driven responses across all units for with and without movement trials are nearly identical in both monkeys (**a**, **b**, left). When the average responses are aligned on the onset of the movement (**a, b** right) if movements were present (blue), or for matched stimulus conditions without movement (orange), the responses on epochs with movement are very similar to corresponding epochs those without movement. It indicates that there was no systematic modulatory effect relative to the movement onset across the population of units. (**c**) We also computed a movement modulation index (*MI*, see Methods) for each unit. On average, the *MI* in each area was close to 0 (mean ± standard deviation; *MI* = -0.009 ± 0.029 p = 10^−5^; −0.011 ± 0.036, p = 10^−5^, −0.008 ± 0.032, p = 10^−4^; for V1, V2, V3/V3A, respectively; two-sided signrank test for deviations from 0, corrected for multiple comparisons); identical scale of the x-axis as in Fig. 3 to facilitate the comparison of the effect sizes. This model-free analysis therefore confirms our model-based analysis and shows that any modulation by the animals’ own movements in these visual areas is very small.

A model-free analysis that relied on neither the singular vector decomposition of the video recordings nor the linear regression confirmed the minimal modulation of the visual activity in V1, V2 and V3/V3A by the animals’ spontaneous movements (Extended Data Fig. 7). For this analysis we computed a movement modulation index (MI; Methods) that was analogously devised to indices that quantify modulation by cognitive states such as attention (see below). It therefore allowed us to contextualize the size of the modulatory effect by movement and compare it directly with the known modulatory effects by cognitive factors.

### Attentional modulation is not associated with modulation by movements

**Fig. 3:**
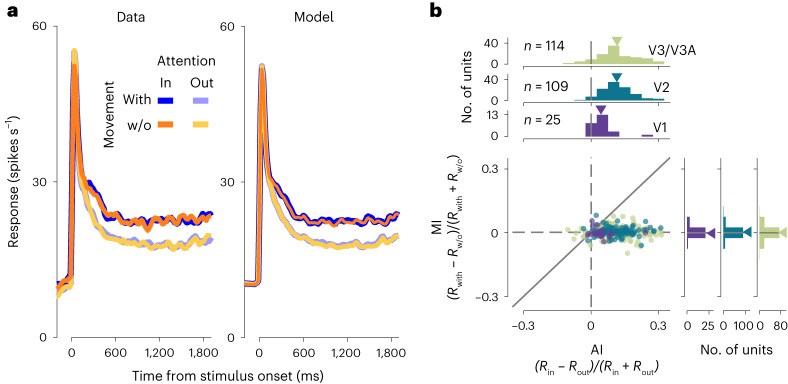
Modulation by spatial attention is not associated with modulation by movement. **a**, Average stimulus-driven responses across all units (*n* = 248; left, peristimulus SDF; right, rates predicted by the full model) separated by attention and the presence or absence of the animals’ spontaneous movements. w/o, Without. **b**, Modulation indices for attention (AI, abscissa*)* are not correlated with those for movement (MI, ordinate) in V1, V2 and V3/V3A. Source data

Modulation by locomotion in mice shows parallels to the modulation by spatial attention in primates^28,29^. Therefore to test for a potential relationship between neural modulation by spontaneous movements and attention, we trained one animal to perform a visual discrimination task while manipulating spatial attention. We then recorded neural activity in the visual cortex while the animal performed this task, and monitored the animal’s own movements. We observed the characteristic^30,31^ increase in neural response when the animal’s attention was directed to the receptive fields (that is, spatial attention), including the typically modest modulation in V1 (ref. ^32^) (Fig. 3a). This attentional modulation, quantified as the attention index (AI), analogous to MI, was not correlated with modulation by spontaneous body movements (Fig. 3b; *P* > 0.3 for all areas; neural modulation by spatial attention was also not correlated with the absolute value of the neural modulation by face/body movements, *P* > 0.2 for all areas). The analysis in Fig. 3b was model free and showed that modulation by the animal’s own movement was about an order of magnitude smaller than the modulation by spatial attention (mean ± s.d., MI = −0.005 ± 0.026, 0.006 ± 0.019, 0.001 ± 0.03; AI = 0.05 ± 0.05, 0.11 ± 0.07, 0.11 ± 0.09 for V1, V2 and V3/V3A, respectively; the distributions for MI versus AI differed significantly in all areas: *P* = 0.003, *P* = 10^−36^ and *P* = 10^−24^ for V1, V2 and V3/V3A, respectively; Student’s *t*-tests, corrected for multiple comparisons). These findings corroborate our model-based results and, as a secondary finding, suggest no association between a modulation by an animal’s own movements and the modulation by spatial attention in macaques.

## Discussion

The present results show that, in macaque monkeys, spontaneous body and face movements accounted for very little of the variability of single-trial neural dynamics in macaque V1, V2 and V3/V3A. This contrasts with results in mice where substantial modulation of visual cortical activity is associated with the animal’s own spontaneous movements^20–22^. The difference in results across species cannot be attributed to a difference in the experimental set-up (compare ref. ^21^) or analysis methods: the present analysis was designed to replicate the approach used in mice (Methods) and, when retinal input in the macaque monkeys was uncontrolled, spontaneous movements did account for appreciable neural variability, as in mice. Our control analyses showed that during trials with more body movements these accounted for an even smaller percentage of neural activity in the macaques than on other trials (Extended Data Fig. 4). It is difficult to compare the degree of movement directly across both species. But this control indicates that, even if it were the case that the macaques moved less than the mice, a difference in the degree of movement would not account for the difference in the observed neural modulation by movements. Moreover, the neural measurements used in the present study recovered the expected modest levels of neural modulation caused by spatial attention^30–32^, even in V1, implying sufficient sensitivity of the neural recordings. Furthermore, our results parallel recent observations in marmosets of a quantitative difference in the neural modulation in visual cortex with locomotion between primates and rodents^33^.

Our results are also consistent with known modulations of visual cortical activity by eye movements, including microsaccades^34–42^ or gaze position^43,44^, but reveal that even these are small (Fig. 2 and Extended Data Figs. 5 and 6) compared with the overall response variability in the macaque visual cortex, in line with early reports^45^. The results in the present study combined with recent findings in marmosets^33^ therefore suggest that decades of neurophysiological research on the primate visual system need not be revisited in light of the pronounced modulations by spontaneous movements observed in mice.

Indeed, in mice, the variance uniquely explained by uninstructed movements in the visual cortex far exceeded that explained by the task and stimulus^21^. In contrast, the data from the macaques in the present study show almost no modulation explained by movements once eye movements have been controlled for. The effects by movements are about an order of magnitude smaller than the known modest modulation by spatial attention. Moreover, the variance uniquely explained by the movements, even when combined with that by slow drifts, was smaller by nearly an order of magnitude compared with the variance explained by the task and stimulus (approximately 1/8; Fig. 2f), the opposite pattern to that in mice.

The linear multivariate regression analysis provides several advantages for the present study. First, using an identical analytical approach to that previously used in mice allows for a direct comparison between studies. Second, it leverages these previous works’ innovations such as the measure of unique variance, as well as their ability to use optimized ridge regression^46^. Third, beyond capturing linear effects, linear regression in this context can be very sensitive and, given the non-negative spike counts within individual time bins, nonlinear interactions between variables (such as gain^9^) will have a linear component. This is facilitated by our incorporation of stimuli into the model using ‘one-hot’ regressors (Methods), resulting in a different weight for each individual stimulus value. This made the model sensitive to nonlinear interactions and can capture, for example, nonlinear tuning to disparity and contrast.

As the spontaneous movements are not controlled by the experimenter, they might be correlated with each other or with other task variables. Model-based analysis is therefore necessary to attribute their effects to specific regressors. It is precisely for this reason that the model-free analysis required careful conditioning on identical stimuli and task parameters (Methods). But this model-free analysis corroborated our main observation that modulation by movements was very small and supports the conclusion that the linear regression approach would have detected a broad range of significant modulatory effects by movements.

The present study exemplifies the challenge of attributing the sources of neural variability when the possible sources themselves are correlated. We disentangled the contribution of the change in the retinal input from the eye movements causing this change by inferring the retinal input during the eye movements. This allowed us to attribute the modulation to predominantly the change in retinal input, that is, the feed-forward sensory input, rather than the movement itself. The challenge of attributing neural variability to its underlying causes is highlighted by another recent study in mice examining modulation associated with multisensory (auditory) stimulation in visual cortex^47^. This study concluded that modulation initially interpreted as multisensory (auditory) seemed instead to be driven by state changes associated with the animals’ movements in response to the auditory stimulus. The findings of both studies are thus complementary within a common theme, which is to identify the sources of neural variability in the visual cortex.

The present study shows that activity in the primate visual areas is only minimally modulated by the animal’s own body movements. Of course, such effects on neural activity are expected to be more pronounced in brain areas with a stronger role in sensorimotor processing. Indeed, recent work reported modulation by spontaneous body movements in the macaque prefrontal cortex that were comparable in size to the animal’s instructed movements^48^. How the degree of modulations by an animal’s own movements change when ascending the visual hierarchy beyond the areas examined here, and how this depends on the type of behavior or behavioral syllables^49^, will require future studies.

Although the results of the present study raise the possibility that some fraction of the neural modulation associated with movement observed in mice in visual areas is related to uncontrolled retinal input, there are good reasons to suspect genuine differences in the mechanisms of embodiment between mice and monkeys. Primates and rodents differ not only in body anatomy but also in behavior and brain organization. Primary visual cortex in the mouse receives substantial direct projections from premotor areas^50^ but does not in the monkey^51^, and the neuromodulatory system in the visual cortex differs in the two species^52,53^. A direct modulation of visual cortical responses by movement may be evident in higher visual areas in primates, which are perhaps a closer analog of primary visual cortex in rodents^54–56^. The difference in results between mice and primates may therefore reflect corresponding differences in anatomy and behavior^57,58^. Primates must make sense of the statistics of their visual input and how that input is shaped not only by their body’s own locomotion^52^ but also prominently by their eye movements^59–61^. These demands may have selected mechanisms to emphasize embodiment that exploit input provided by the eyes themselves.

## Methods

### Animals

Two adult male rhesus monkeys (*Macaca mulatta*) were used as subjects (animal 1 (M1); animal 2 (M2); 21 and 13 years old, respectively, each 9 kg). All protocols were approved by the National Eye Institute Animal Care and Use Committee (M1; animal study proposal NEI-567) or by the relevant local authority (M2; protocol CIN 1/14), the Regierungspräsidium Tübingen, Germany, and all experimental procedures were performed in compliance with the US Public Health Service Policy on Humane Care and Use of Laboratory Animals. Under general anesthesia, the monkeys were surgically implanted with a titanium head post and in a subsequent procedure with a recording chamber (19-mm inner diameter, cilux, Crist Instrument) over right hemispheric V1 (M1), and with two titanium recording chambers (25-mm inner diameter) over the operculum of V1 on both hemispheres (M2), guided by structural magnetic resonance imaging of the brain.

### Behavioral tasks

#### Visual fixation

Animal M1 was required to fixate on a small spot (fixation point (FP), 0.3° diameter) at the center of the screen for about 2 s to receive a liquid reward, while a drifting sinusoidal luminance grating was flashed four times (450-ms duration each separated by an interval of approximately 50 ms of a blank screen) over the receptive fields (RFs) of the recorded units (Fig. 1b, left). In addition to visual fixation, animal M2 also performed a visual discrimination task.

#### Disparity discrimination

Animal M2 performed a disparity discrimination task (Fig. 1b, right) previously described in detail^62^. Briefly, once the animal fixated on an FP (0.1° diameter), two circular dynamic random-dot stereograms (RDSs; for details, see ‘Visual stimuli’), consisting of a disparity-varying center surrounded by an annulus fixed at zero disparity, were presented, one in each visual hemifield. Stimuli presented in one hemifield were task relevant. The animal had to judge whether the center disparity of the relevant RDS was protruding (‘near’; negative disparity) or receding (‘far’; positive disparity) relative to a surrounding annulus. After 2 s, the FP and the RDSs were replaced with two choice icons (circular RDSs at 100% disparity signal, one at the near, the other at the far signal disparity) positioned above and below the FP but horizontally offset toward the cued side. The animal was rewarded after making a saccade within 2 s of the onset of the choice icons, to the choice icon that had the same sign of disparity signal as the stimulus. The task-relevant hemifield was cued by three instruction trials at the beginning of each 50-trial block. On instruction trials a single stimulus was presented on the task-relevant side. The vertical position (~3° above or below the FP) of the choice icons was randomized across trials to prevent a fixed mapping between the chosen disparity sign and saccade direction.

### Visual stimuli

Visual stimuli were back-projected on a screen (Stewart Filmscreen) by a DLP LED projector (PROPixx, VPixx Technologies; 1,920 × 1,080 pixel^2^ resolution). The display was achromatic and the luminance steps were linearized (mean luminance: 72 cd m^−2^ for M1, 30 cd mm^−2^ for M2). Visual stimuli were presented on a uniform display at the mean luminance. Separate images were delivered to the two eyes (120 Hz for M1 and 60 Hz for M2, for each eye) using a combination of an active circular polarizer (DepthQ, Lightspeed Design Inc.) in front of the projector and two passive circular polarizers with opposite polarities (American Polarizers) in front of the eyes. The viewing distance was 45 cm for M1 and 97.5 cm for M2, at which the display subtended 74° by 42° for M1 and 32° by 18° for M2.

Stimuli used in the fixation task for M1 were drifting circular sinusoidal luminance gratings, the position and size of which were tailored to the collective RFs of the recording site. The spatial frequency was adjusted inversely proportional to the RF size (0.25–1.5 cycle per ° with a mean of 0.64 cycle per ° averaged across recording sessions) and the temporal frequency was either 4 or 5 Hz. The mean diameter of the stimulus was 3.5° (range, 1.5–6.0°) for V1 on the operculum, 4.7° (range, 3.5–6.0°) for V1 in the calcarine sulcus, 5.1° (range, 2.5–8.0°) for V2 and 7.9° (range, 6.0–12.0°) for V3. The contrast of the stimulus during each of four 450-ms stimulus epochs on a trial was randomly chosen from four values (0, that is, blank stimulus, 6.25, 25 and 100% of the maximum contrast) with equal probabilities.

Stimuli used in the disparity discrimination task for M2 were circular dynamic RDSs (50% black, 50% white dots, dot size typically 0.08° radius, 50% dot density) with a disparity-varying central disk (3–5° in diameter, approximately matching the RF size of the recorded units) surrounded by an annulus of zero disparity (1° width). The positions of the dots were updated on each frame. The central disk consisted of signal frames randomly interleaved with noise frames. For each session, the signal disparities (one near disparity, one far disparity) were fixed. The center disparity of the stimulus was updated on each video frame. On ‘signal frames’, the center disparity was one of the signal disparities, held constant across each trial. On a ‘noise frame’, the disparity of the center disk was randomly chosen from a uniform distribution of 9 values equally spaced from −0.4° to 0.4°. The task difficulty on a trial was defined as the ratio of the signal to noise frames such that 100% means that all frames were signal frames and 0% that all frames were drawn from the noise distribution. On a 0% trial, the reward was randomly given 50% of times. The choice target icons were also circular RDSs but slightly smaller than the stimuli and always presented at 100% near and far signal. We assessed disparity tuning before the behavioral task in separate visual fixation experiments using RDSs (450-ms duration), with a disparity that varied typically from −1° to 1° in 0.1° increments. The two signal disparities in each session were chosen to approximately match the preferred and nonpreferred disparities by most of the recorded units.

Receptive fields (Extended Data Fig. 1) of the recorded units were first approximated by a bar stimulus with an orientation and position that were manually controlled, then quantitatively measured with strips of horizontal or vertical bars (450-ms duration each, typically white and black bars but sometimes RDSs at the preferred disparity when they evoked stronger responses) that were equally spaced over the range covering RFs estimated by manual sweeping (typically 9–11 positions with intervals were determined by the collective RF range).

Visual stimuli were generated in MATLAB (MathWorks) by customized code^63^, adapted from Eastman and Huk^64^ using the Psychophysics toolbox^65^.

### Electrophysiological recordings

Extracellular recordings were made from areas V1, V2 and V3/V3A using multichannel laminar probes (Plexon Inc.; V/S Probes, 24/32 channels, 50–100 μm intercontact spacing). Neuronal signals were amplified, filtered (250 Hz to 5 kHz) and digitized (30-kHz sampling rate) by the Grapevine Neural Interface Processor (NIP, Ripple Neuro) run by the Trellis software (Ripple Neuro) that interfaced with MATLAB via Xippmex (v.1.2.1; Ripple Neuro).

We inserted recording probes on each day of experiments via the operculum of V1 using a custom-made (M1) or customized (M2; NaN Instruments) micro-drive placed approximately normal to the surface. We initially mapped the recording sites using single tungsten-in-glass electrodes (Alpha Omega) to determine the RF locations and assess the selectivity for horizontal disparity. During data collection, visual areas were identified using two physiological criteria: (1) transitions from gray matter to white matter, which was typically characterized by a silent zone that spanned a few consecutive channels showing weak or no visually driven responses; and (2) abrupt shifts in the RF location and size and often abrupt changes in the tuning preferences for orientation or disparity. Final assignments of channels to visual areas were done offline with the aid of RF maps constructed from RF location and size determined from quantitative fitting (see below) across all sessions (Extended Data Fig. 1), combined with the structural MR scans. As a result of the similarity between the disparity selectivity in V3 and V3A^66^, we did not seek to further assign channels to V3 or V3A, and instead designated them collectively as V3/V3A.

On each day of experiments, after the laminar probe had been advanced to a depth at which most channels spanned the visual area from which we intended to record, we usually advanced it further to confirm the visual area underneath. Then, we withdrew the probe back to the desired depth and waited for at least 30 min before data collection to allow time for the tissue around the probe to be stabilized, thereby minimizing vertical drifts of the recording site along the probe. After we mapped the RFs, we evaluated the orientation tuning of the units using drifting gratings presented over the RFs. Occasionally, the tuning for spatial frequency and temporal frequency was also probed using drifting gratings with orientation set to the one preferred by most of the units. The parameters of the stimulus used in the subsequent main experiment were then determined based on the assessed tuning properties. When the probe picked up the responses of units from multiple areas (for example, V1 and V2 or V2 and V3A), we tried to set the location and size of the stimulus to cover the RFs of the units from both areas, which was usually successful. We checked the RF locations across channels after, and sometimes in between, data collection to diagnose vertical drifts of the neural tissue relative to the electrode channels. We only included units that remained in the same visual areas during the entire data collection period and excluded units with activity picked up by channels positioned within the transition depth between visual areas at any time during data collection.

### Measurements of eye position

We monitored the animals’ binocular eye positions and pupil size using the EyeLink 1000 infrared video tracking system (SR Research) at a sampling frequency of 500 Hz.

### Recording of face and body movements

To record the face and body movements of the animals during data collection, we installed infrared (940 nm) light-emitting diodes and at least two cameras (Fig. 1a; M1—Stingray camera integrated in a CinePlex Behavioral Research System, Plexon Inc., 60- or 80-Hz sampling rate, downsampled to 20 Hz and spatially downsampled by 2 × 2 pixels^2^ for analysis; M2—Imaging Source DMK camera; triggered image acquisition at 12.5 Hz), one pointing to the face and one to the front view of the body.

### Data analysis

#### Spike sorting

We sorted spikes from single- or multi-units offline using Kilosort2.5 (ref. ^67^) followed by manual curation in Python (www.github.com/cortex-lab/phy) for data from M1 and using the Plexon Offline Sorter (v.3.3.5; Plexon Inc.) for data from M2. We analyzed spikes from both single- and multi-units isolated by the spike-sorting procedures, which we refer to as units without distinction.

### Receptive fields

To measure RFs, we averaged the multi-unit response (spike count during stimulus interval) on each recording channel for each position of the bar stimuli. We fitted a Gabor function to the mean response as a function of stimulus position, separately for the horizontal and vertical dimensions, using MATLAB (lsqnonlin). The center of the RF was defined as the position at the peak of the fitted function and the width as the distance between the two positions flanking the peak at which the fitted function reached 20% of its peak above the offset (Extended Data Fig. 1).

### Motion decomposition

To quantify the face and body movements, we selected regions of interest (ROIs) from the videos with the face view and the frontal body view to include only the animal’s face and body. The movements in the selected ROIs were decomposed into movement components using SVD following the method in ref. ^20^ (www.github.com/MouseLand/FaceMap), which builds on previous work^68^, via temporal-segment-wise SVD (~1-min-long segments of the videos; Fig. 1c). The motion matrix *M* of the video, where *M* is the absolute pixel-wise difference between two consecutive frames (no. of the pixels in the ROI × no. of video frames − 1), was then projected on to the first 1,000 movement components to calculate their temporal profiles. These temporal profiles correspond to the face/body movement regressors used in the ridge regression-modeling approach described below. To evaluate the contribution of the movement components of the eye region in the face view to neural modulation (Results), we performed the same SVD analysis on the face videos after the eye regions were removed from the face ROI.

### Modeling neural activity during trials

We modeled the spiking activity of each unit as a linear combination of task-related and task-unrelated events within a session using ridge regression adapted after Musall et al.^21^. Our linear multivariate regression is thus analogous to the approach used previously in mice^21,22^ and benefits from its many advantages, as described in the main text above. Although a nonlinear model might achieve better overall predictions, it would not reveal pronounced modulation by movements where a linear model would detect none. We therefore used a linear statistical model to facilitate this comparison to the results from mice. Note that our regression procedure differed explicitly from the original application in mice in two respects (as explained in detail below): (1) we segmented training and testing (crossvalidation) sets based on continuous blocks to avoid bleed-through of slow-timescale correlations; and (2) we included additional drift regressors in the model to prevent it from using other regressors to explain instabilities in the recordings or state of the animal that manifests over time scales of the order of >100 trials.

Regressors for task-related events reflect the stimulus, the time since the start of the trial, the timing of reward in both animals and, in addition, the presence of choice targets and saccadic choice in animal M2 (Fig. 2a). Regressors for task-unrelated events were based on face and body movements, and a slow drift term to capture nonstationarities in firing rates of each unit. Below we describe the individual regressors.

#### Stimulus regressors

These were discrete binary vectors with one dimension for each distinct stimulus (that is, disparity and contrast). They had a value of 1 for the appropriate stimulus dimension in the time periods spanning the stimulus presentation window and 0 elsewhere. Separate regressors were used to model different stimulus values (one-hot stimulus regressors). In addition, in animal M1, within each stimulus value (that is, the four contrast values), separate regressors were used for the four successive samples in time (Extended Data Fig. 2). In animal M2, within each stimulus value (that is, the disparity value on each video frame), separate regressors were used for stimulus presentations on the left and right hemifields (that is, whether the attended stimulus was within or outside the RF of the recorded neuron). This one-hot regressor design allowed us to capture modulation of spiking activity as a function of sample position within the stimulus sequence and stimulus contrast in animal M1 and as a function of disparity and attended location in animal M2.

#### Reward regressors

These were discrete binary vectors with a value of 1 at reward onset and 0 elsewhere.

#### Time regressors

These were discrete binary vectors with a value of 1 at stimulus onset and 0 elsewhere and were used to model modulations in spiking activity owing to stimulus onset and offsets (Fitting procedure).

#### Choice target, binary choice and choice saccade regressors

Animal M2 performed a discrimination task requiring it to make a saccade to one of the two targets presented after the stimulus offset. Target regressors were discrete binary vectors with a value of 1 at target presentation and 0 elsewhere. Separate regressors were used to model targets presented offset to the left and right hemifields. Binary choice regressors were discrete regressors with a value of ±1 to model near versus far disparity choices when the animal reported a choice and 0 elsewhere. Choice saccade regressors were discrete regressors with a value of ±1 to model saccades to the top and bottom target when the animal reported the choice and 0 elsewhere.

#### Drift regressors

Nonstationarity in firing rates for each unit was modeled as a set of analog regressors using tent basis functions spanning the entire session^62^. These basis functions allow for a smoothly varying drift term that can be fitted as linear model terms. We defined anchor points placed at regular intervals within each session (ten and eight anchor points for animals M1 and M2, respectively), each denoting the center of each basis function. The basis function has a value of 1 at the corresponding anchor point and linearly decreases to 0 at the next, and previous, anchor point and remains 0 elsewhere. Thus, any offset at each time point due to slow drift in firing rate is modeled by a linear combination of the two basis functions. Although the drift regressors were included to account for nonstationarities related to experimental factors, they would also capture factors related to slowly changing cognitive states throughout a session^69^. To therefore avoid the drift predictors accounting for the block-wise alternation in spatial attention for M2, we ensured that no more than one anchor point was used for each pair of successive, alternating blocks of attention.

#### Face and body movement regressors

**Extended Data Fig. 8:**
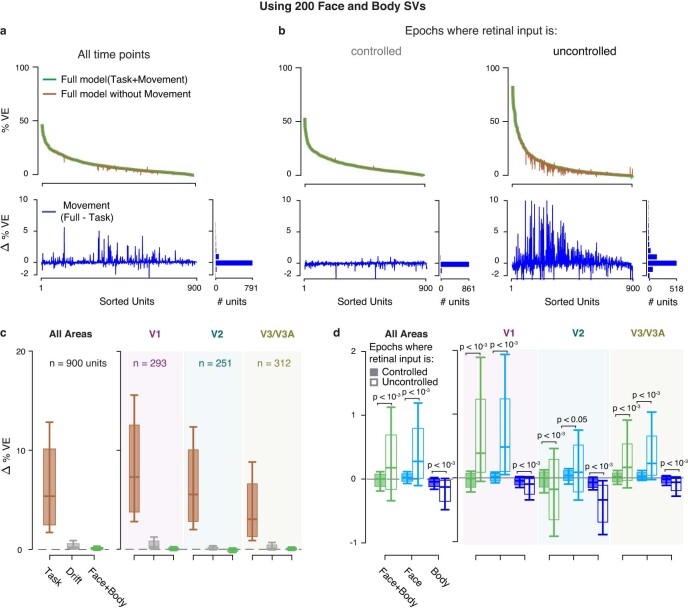
Linear encoding model fits, using 200 face and body components. **a–d**: Format as in Fig. 2d–g. Increasing the number of SV components from face and body videos in the linear encoding model to 200 from 30 (Fig. 2) did not increase the variance explained by spontaneous movements. Box plots, inter-quartile range; whiskers, range covering 66 percentile of the data; solid lines inside the box plots, median across units. P-values obtained using two-sided permutation test, uncorrected for multiple comparisons. **c**, **d**: Data for V1, V2, V3/V3A are shaded in purple, green and brown, respectively.

The temporal profiles of the top 30 SVD components (SVs) of videos capturing movements in the face and body regions in both animals were used as analog regressors to model modulation in spiking activity owing to movements. Note that, as we did not additionally include regressors for pupil size or eye position in the main analysis (Extended Data Fig. 5), this gives the included movement regressors the possibility of also explaining neuronal variability that might otherwise be explained by pupil regressors as a result of the correlation between these covariates^17^. To avoid overfitting, we limited our analysis to 30 SVs, but our results were qualitatively similar when the top 200 SVs were used instead (Extended Data Fig. 8).

#### Eye and pupil regressors

Time courses of eye movements and pupil size recorded using the eye-tracking camera were used as analog regressors in a control analysis. Across both eyes we computed time-varying kernels of the mean horizontal and vertical eye movements, mean eye speed obtained by computing the first derivative of eye movements along the time axis, mean pupil size and mean of the first differential of pupil size along the time axis.

### Fitting procedure

Recordings from each session were first split into individual trials. We modeled only successfully completed trials. The resulting mean number of trials across sessions was 1,820 (that is, 7,280 presentations for each stimulus contrast) for M1 (range, 219–3,444) and 662 for M2 (range, 617–713). Each trial was defined by a 300-ms pre-stimulus period, the stimulus presentation window and a 1,000-ms window after stimulus offset. This allowed us to split time periods within an individual trial into those where the retinal input was controlled, that is, the animal maintained visual fixation, and those where the retinal input was uncontrolled. Time points within each session were discretized into nonoverlapping 16.67-ms-wide time bins, matching the lower framerate of the stimulus displays used for the two monkeys. Spiking activity of each unit was quantified as the number of spikes in each time bin, and all the regressors were downsampled to 60 Hz while preserving their discrete/analog nature. In trials where the 1-s post-stimulus window of the current trial overlapped with the 0.3-s pre-stimulus window of the next trial, we reduced the post-stimulus window to include only the nonoverlapping time bins. In the control analysis with eye and pupil regressors (Extended Data Fig. 6), we excluded those time points where the signals from the eye tracker were undefined (for example, during blinks or when the eye position moved outside the range that the eye tracker could monitor) during model fitting.

As the effect on neural activity of a given regressor will often play out across time, we modeled the effect of each regressor using a time-varying ‘event kernel’ by creating numerous copies of individual regressors, each shifted in time by one frame^21^ relative to the original using predefined time windows. These time windows for stimulus, reward and choice target regressors were 250 ms post-event, for choice saccade regressors 500 ms pre- and post-event and for time regressors spanned the entire duration of the trial after the stimulus onset, including the post-stimulus window. The time-varying kernels of the analog movement regressors were modeled by convolving the temporal profiles of the corresponding component with separate tent basis functions with anchor points at −100 ms, 0 ms and 100 ms with respect to the movement event. This allowed us to capture the temporal dependence of spiking activity on the movement within a 400-ms time window, resulting in a total of 90 regressors each for face and body movement components. Similarly, the time-varying kernels of horizontal and vertical eye movements, eye speed, pupil size and pupil size derivative regressors were obtained by convolving the regressors with separate tent basis functions with anchor points at −50 ms, 0 ms and 50 ms, capturing the temporal dependence of spiking activity within a 200-ms time window. All the event kernels were constructed at the level of individual trials.

We fitted the models using ridge regression and tenfold crossvalidation across trials to avoid overfitting^70^. Trials were randomly assigned to training or test dataset within each fold, such that no event kernel spanned samples from both the training and the test datasets. Separate ridge penalty parameters were estimated for each unit during the first crossvalidation fold, which were then used in subsequent folds. All the regressors were normalized to have zero mean and unit variance before fitting, such that penalization owing to the ridge parameter was similar across all regressors and ensured that all regressors had a fair chance of explaining the data. Furthermore, our trial definition coupled with the tenfold crossvalidation resulted in the test data containing a combination of different stimulus intensities as well as controlled and uncontrolled retinal input epochs.

### Model performance

We used %VE as the measure of model performance. This is computed based on the variance of the residual of the model prediction (prediction − the binned spike count) compared with the overall variance of the observed binned data. Note that %VE at the single-trial level at these time resolutions (16-ms bins) is dominated by spike count variability, and the same models that explained, on average, 94% of the variance in the SDF averaged across trials (Fig. 2c) explain a mean of 9.8 %VE (Fig. 2d). Furthermore, to determine the ‘unique’ effect of different task-related and task-unrelated events on the spiking activity, we estimated the ‘unique variance’ as defined by Musall et al.^21^. This metric was devised to account for the fact that many predictors in the model are correlated. It is the variance, explained by each class of regressors by computing the %VE for a reduced model obtained through shuffling in time only the regressors under consideration, leaving all the others intact and subtracting this from the %VE of the full model. Note that, by shuffling rather than eliminating a given regressor, the resulting model will have the same number of parameters as the full model and, thus, if the regressor contained no additional (or ‘unique’) information to predict the neural response, it would result in the same %VE. The resulting difference (∆%VE) thus gives a measure of the predictive power unique to each regressor^21^.

### MI and AI

To determine periods with movement (Fig. 3), we used the motion matrix *M* (Motion decomposition) for the face and body, where *M* is the absolute pixel-wise difference between two consecutive video frames (no. of the pixels in the ROI × no. of video frames − 1). We then averaged *M* over pixels to compute the average motion versus time (1 × no. of frames − 1). Periods with movement were defined as those when the average motion exceeded the 80th (for M2: 90th) percentile across all time points in either the face or the body view, whereas periods without movement were defined as those for which the average motion was below its median (for M2: below the 20th percentile) across all time points, in either the face or the body view. (We note that we confirmed that the results were qualitatively similar when we used only the body view or only the face view, indicating that neither type of movement had a sizable effect on the MI.) To reduce the possibility that differences in activity between movement epochs and without movement epochs were driven by systematic stimulus differences between the two sets (for example, driven by systematic associations between the timing of movements and certain stimuli), we conditioned epochs assigned to movement epochs and without movement epochs such that the stimulus conditions were matched. For M1, we matched: (1) the lag between the stimulus onset and movement onset, or between stimulus onset and a corresponding no-movement period (to within 10 ms); (2) the contrast of the stimulus; (3) the position of this stimulus in the trial (‘sample’, see Extended Data Fig. 2); (4) the contrast of the immediately preceding stimulus; and (5) the interstimulus interval (to within 50 ms). For M2 we matched: (1) the lag between the stimulus onset and movement onset, or between stimulus onset and a corresponding no-movement period (to within 10 ms); (2) the stimulus type, that is, disparity, signal strength and attention condition; and (3) the intertrial interval (to within 300 ms). We calculated the MI and the AI based on the average spike rates (*R*; computed after removing nonstationarities across the recording session using the drift term of the linear regression model described above) from 0.15 s to 0.45 s after stimulus onset for M1 and 0.15 s to 2 s after stimulus onset for M2, as:

$${{\rm{MI}}}=\frac{{R}_{{{\rm{with}}\; {\rm{movement}}}}-{R}_{{{\rm{without}}\; {\rm{movement}}}}}{{R}_{{{\rm{with}}\; {\rm{movement}}}}+{R}_{{{\rm{without}}}\;{{\rm{movement}}}}}{\rm{and}}\;{{\rm{AI}}}=\frac{{R}_{{{\rm{attention}}\; {\rm{in}}}}-{R}_{{{\rm{attention}}\; {\rm{out}}}}}{{R}_{{{\rm{attention}}\; {\rm{in}}}}+{R}_{{{\rm{attention}}\; {\rm{out}}}}}.$$

We computed the spike density functions (Fig. 3a and Extended Data Fig. 7) by convolving peristimulus time histograms (16-ms resolution) for each unit with a temporal smoothing function (half-Gaussian function; s.d. 16 ms) and averaging this across units.

### Dataset

**Extended Data Fig. 9:**
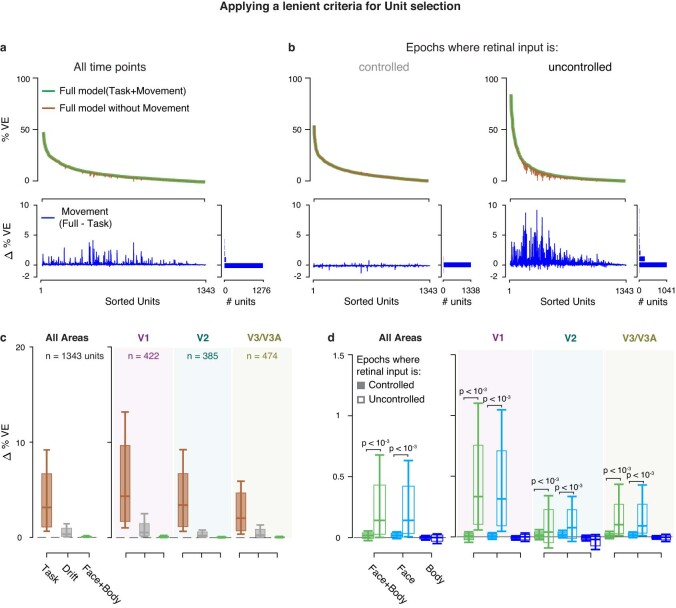
Linear encoding model fits, using a lenient criterion for unit selection. **a–d**: Format as in Fig. 2d–g. We used all units for which the % VE by the full model was > 0 (see Methods). Box plots, inter-quartile range; whiskers, range covering 66 percentile of the data; solid lines inside the box plots, median across units. P-values obtained using two-sided permutation test, uncorrected for multiple comparisons. **c**, **d**: Data for V1, V2, V3/V3A are shaded in purple, green and brown, respectively.

Our dataset consists of a total of 1,407 units: 1,139 units from M1 recorded in 54 sessions and 268 units from M2 recorded in 5 sessions. We excluded 507 units from the analysis that failed to meet the following criteria: (1) a minimum mean firing of 2 spikes s^−1^ during stimulus presentation epochs in each of the four quartiles of the session; and (2) a minimum of 0 %VE of the full model during both retinal input-controlled and -uncontrolled epochs. Among the remaining 900 units, 653 units were from M1 (V1 (269), V2 (143) and V3/V3A (198)) and 247 units from M2 (V1 (24), V2 (108) and V3/V3A (114)). Results were qualitatively similar when the minimum firing rate criterion was relaxed to include 1,343 units in the model (Extended Data Fig. 9). For the model-free analysis in Fig. 3 and Extended Data Fig. 7, we used only the first criterion, avoiding subselection of units based on model fits. We did not assign visual areas to 44 units recorded in 3 sessions from M1 and 1 session from M2, in which the receptive location and size were not consistent with the overall topography of the offline RF map, to unambiguously assign the recording sites, but included them when data were combined across areas.

### Statistics and reproducibility

Sample size of the main dataset was 900 units across visual areas V1, V2 and V3/V3A from two adult male rhesus macaques (*M. mulatta*). Use of data from two rhesus monkeys is typical for comparable neurophysiological studies in macaques. No statistical method was used to predetermine sample size, but the number of units are comparable to recent neurophysiological studies in macaques^62^. Data exclusions are clarified in Dataset. The experiments were not randomized because all comparisons were made within subject across conditions, that is, each subject was its own control. The investigators were not blinded because blinding was not relevant as no group allocation was done in our study.

To avoid the assumption that the data were normally distributed, we used nonparametric permutation tests^71^ to test for group-level significance of individual measures, unless otherwise specified. This was done by randomly switching the condition labels of individual observations between the two paired sets of values in each permutation. After repeating this procedure 10,000×, we computed the difference between the two group means on each permutation and obtained the *P* value as the fraction of permutations whose absolute values exceeded the observed absolute difference between the means. All *P* values reported were computed using two-sided tests unless otherwise specified.

### Reporting summary

Further information on research design is available in the Nature Portfolio Reporting Summary linked to this article.

## Online content

Any methods, additional references, Nature Portfolio reporting summaries, source data, extended data, supplementary information, acknowledgements, peer review information; details of author contributions and competing interests; and statements of data and code availability are available at 10.1038/s41593-023-01459-5.

### Supplementary information

Supplementary Information Supplementary Table 1. Results are robust to varying thresholds of unique variance explained by movements to classify neurons as modulated by movements. Proportion of units for which the unique variance of movements (Fig. 2e) exceeds different thresholds of unique variance. *P* values compare the proportions between controlled and uncontrolled retinal input epochs using a two-sided χ^2^ test, uncorrected for multiple comparisons.

Reporting Summary

Supplementary Video 1 Example video clip showing typical spontaneous movements of one of the animals (M1) during a recording session. The labels of the body parts (obtained using DeepLabCut^72^) are only included for demonstration purposes here but not used for our analysis.

Supplementary Video 2 Example clip from the face video of the animal M2 (top) is shown with the temporal profiles (bottom middle) of four singular vectors (SVs, bottom left and right) synchronized with the video clip. Note that SVs 2 and 7 capture predominantly movements around the eyes whereas SVs 1 and 11 capture movements of the mouth.

Supplementary Video 3 Example in the same format as Supplementary Video 2 but showing a clip from the body video of M1 with temporal profiles of four SVs synchronized with the clip. Although all the illustrated SVs capture movements of the hands, SVs 3 and 2 also capture movements of the legs and feet. The video clip includes that used in Supplementary Video 1.

### Source data

Source Data Fig. 2 Contains the numerical values used to produce individual **b**–**g** as separate sheets.

Source Data Fig. 3 Contains the numerical values used to produce individual **a** and **b** as separate sheets.

## Data Availability

Data used in the present study (10.6084/m9.figshare.21767345) are publicly available. Source data are provided with this paper.

## Code availability

Analysis code (https://github.com/NienborgLab/TalluriKang_et_al_2022) is publicly available.
