# Supplementary material for: Activity in primate visual cortex is minimally driven by spontaneous movements
Source: Nat Neurosci. 2023 Oct 12;26(11):1953–9. doi: 10.1038/s41593-023-01459-5 (PMC10620084; doi:10.1038/s41593-023-01459-5)
Supplement: Supplementary file 1 — Supplementary Table 1. Results are robust to varying thresholds of unique variance explained by movements to classify neurons as modulated by movements. Proportion of units for which the unique variance of movements (Fig. 2e) exceeds different thresholds of unique variance. P values compare the proportions between controlled and uncontrolled retinal input epochs using a two-sided χ2 test, uncorrected for multiple comparisons. [file 41593_2023_1459_MOESM1_ESM.pdf]

nature neuroscience

Article

https://doi.org/10.1038/s41593-023-01459-5

Activity in primate visual cortex is minimally driven by spontaneous movements

In the format provided by the authors and unedited

Threshold (% ΔVE)	Controlled Retinal input	Uncontrolled retinal input	P-value (c^2 test)
All areas			
0	549/900; 61%	768/900; 85%	$< 10^{-16}$
0.05	172/900; 19%	697/900; 77%	$< 10^{-16}$
0.1	48/900; 5%	606/900; 67%	$< 10^{-16}$
0.5	1/900; 0.1%	252/900; 28%	$< 10^{-16}$
1	0/900; 0%	125/900; 14%	$< 10^{-16}$
V1			
0	159/293; 54%	281/293; 96%	$< 10^{-16}$
0.05	42/293; 14%	268/293; 91%	$< 10^{-16}$
0.1	15/293; 5%	246/293; 84%	$< 10^{-16}$
0.5	1/293; 0.3%	132/293; 45%	$< 10^{-16}$
1	0/293; 0%	67/293; 23%	$< 10^{-16}$
V2			
0	160/251; 64%	168/251; 67%	0.45
0.05	59/251; 24%	145/251; 58%	10^{-15}
0.1	16/251; 6%	131/251; 52%	$< 10^{-16}$
0.5	0/251; 0%	35/251; 23%	10^{-9}
1	0/251; 0%	15/251; 6%	10^{-4}
V3/V3A			
0	211/312; 68%	277/312; 89%	10^{-10}
0.05	64/312; 20%	242/312; 78%	$< 10^{-16}$
0.1	13/312; 4%	191/312; 61%	$< 10^{-16}$
0.5	0/312; 0%	61/312; 20%	10^{-16}
1	0/312; 0%	28/312; 9%	10^{-9}

Supplementary Table 1

Results are robust to varying thresholds of unique variance explained by movements to classify neurons as modulated by movements. Proportion of units for which the unique variance of movements (Fig **2E**) exceeds different thresholds of unique variance. P-values compare the proportions between controlled and uncontrolled retinal input epochs using a two-sided chi-square test, uncorrected for multiple comparisons.
