# Supplementary material for: Activity in primate visual cortex is minimally driven by spontaneous movements
Source: Nat Neurosci. 2023 Oct 12;26(11):1953–9. doi: 10.1038/s41593-023-01459-5 (PMC10620084; doi:10.1038/s41593-023-01459-5)
Supplement: Supplementary file 2 — Reporting Summary [file 41593_2023_1459_MOESM2_ESM.pdf]

nature portfolio

Corresponding author(s): Henrikje Nienborg
Last updated by author(s): Aug 18, 2023

Reporting Summary

Nature Portfolio wishes to improve the reproducibility of the work that we publish. This form provides structure for consistency and transparency in reporting. For further information on Nature Portfolio policies, see our Editorial Policies and the Editorial Policy Checklist.

Statistics

For all statistical analyses, confirm that the following items are present in the figure legend, table legend, main text, or Methods section.

n/a	Confirmed	
☐	☒	The exact sample size (*n*) for each experimental group/condition, given as a discrete number and unit of measurement
☐	☒	A statement on whether measurements were taken from distinct samples or whether the same sample was measured repeatedly
☐	☒	The statistical test(s) used AND whether they are one- or two-sided<br>*Only common tests should be described solely by name; describe more complex techniques in the Methods section.*
☐	☒	A description of all covariates tested
☐	☒	A description of any assumptions or corrections, such as tests of normality and adjustment for multiple comparisons
☐	☒	A full description of the statistical parameters including central tendency (e.g. means) or other basic estimates (e.g. regression coefficient) AND variation (e.g. standard deviation) or associated estimates of uncertainty (e.g. confidence intervals)
☐	☒	For null hypothesis testing, the test statistic (e.g. *F*, *t*, *r*) with confidence intervals, effect sizes, degrees of freedom and *P* value noted<br>*Give P values as exact values whenever suitable.*
☒	☐	For Bayesian analysis, information on the choice of priors and Markov chain Monte Carlo settings
☒	☐	For hierarchical and complex designs, identification of the appropriate level for tests and full reporting of outcomes
☒	☐	Estimates of effect sizes (e.g. Cohen's *d*, Pearson's *r*), indicating how they were calculated

Our web collection on statistics for biologists contains articles on many of the points above.

Software and code

Policy information about availability of computer code

Data collection	Visual stimuli used in the experiments were generated in MATLAB (MathWorks, Natick, MA) by custom-written code based on PLDAPS (Eastman and Huk, 2012) and the psychophysics toolbox-3 (https://github.com/Psychtoolbox-3/Psychtoolbox-3); neuronal signals collected using multichannel recording probes were amplified, filtered and digitized by the Grapevine Neural Interface Processor (NIP, Ripple Neuro, Salt Lake City, UT) run by the Trellis software (v1.4.3, Ripple Neuro, Salt Lake City, UT) that interfaced with MATLAB via Xippmex (v1.2.1; Ripple Neuro, Salt Lake City, UT); animals' binocular eye positions were measured using the EyeLink 1000 infrared video tracking system (SR Research, Ottawa, ON, Canada). Video acquisition during the neurophysiological data collection was done using two cameras, one for the face view, one for the frontal body view: Stingray cameras integrated in a CinePlex Behavioral Research System, Plexon Inc., Dallas, TX (for M1); Imaging Source DMK cameras (for M2).
Data analysis	Spikes from single- or multi-units were sorted using Kilosort2.5 followed by manual curation in Python (www.github.com/cortex-lab/phy) for data from animal M1, and using the Plexon Offline Sorter (v3.3.5; Plexon Inc., Dallas, TX) for data from animal M2. All other data analyses were performed using custom written code in MATLAB (Mathworks, Natick, MA), available at (https://github.com/NienborgLab/TalluriKang_et_al_2022).

For manuscripts utilizing custom algorithms or software that are central to the research but not yet described in published literature, software must be made available to editors and reviewers. We strongly encourage code deposition in a community repository (e.g. GitHub). See the Nature Portfolio guidelines for submitting code & software for further information.

Data

Policy information about availability of data

All manuscripts must include a data availability statement. This statement should provide the following information, where applicable:

- Accession codes, unique identifiers, or web links for publicly available datasets
- A description of any restrictions on data availability
- For clinical datasets or third party data, please ensure that the statement adheres to our policy

Data is available at https://figshare.com/s/c2b2494fba97e7b3c2b1.

Human research participants

Policy information about studies involving human research participants and Sex and Gender in Research.

Reporting on sex and gender	Human participants were not used in the study
Population characteristics	Human participants were not used in the study
Recruitment	Human participants were not used in the study
Ethics oversight	Human participants were not used in the study

Note that full information on the approval of the study protocol must also be provided in the manuscript.

Field-specific reporting

Please select the one below that is the best fit for your research. If you are not sure, read the appropriate sections before making your selection.

☒ Life sciences ☐ Behavioural & social sciences ☐ Ecological, evolutionary & environmental sciences

For a reference copy of the document with all sections, see nature.com/documents/nr-reporting-summary-flat.pdf

Life sciences study design

All studies must disclose on these points even when the disclosure is negative.

Sample size	Sample size of the main dataset was 900 units across visual areas V1, V2, V3/V3a from two adult male rhesus macaques (Macaca mulatta). Using data from two rhesus monkeys is typical for comparable neurophysiological studies in macaques. No statistical method was used to predetermine sample size but the number of units are comparable to recent neurophysiological studies in macaques (e.g. Quinn et al. 2021).
Data exclusions	We used all trials in the analysis for which the animals completed the task, and for which the video recordings of the animals were available. Exclusion from this dataset for our main results are clarified in the section 'Dataset' in the methods, as described also here: Our dataset consists of a total of 1407 units: 1139 units from M1 recorded in 54 sessions and 268 units from M2 recorded in 5 sessions. We excluded 507 units from the analysis that failed to meet the following criteria: (1) a minimum mean firing of 2 spks/s during stimulus presentations epochs in each of the four quartiles of the session, and (2) a minimum of 0 %VE of the full model during both retinal input controlled and uncontrolled epochs. Among the remaining 900 units, 653 units were from M1 (V1 - 269, V2 - 143, V3/V3A - 198) and 247 units were from M2 (V1 - 24, V2 - 108, V3/V3A - 114). Results were qualitatively similar when the minimum firing rate criterion was relaxed to include 1343 units in the model (Supplementary Fig. S8). For the model-free analysis in Fig. 3 and supplementary Fig. S6, we only used the first criterion, avoiding sub-selection of units based on model-fits. We did not assign visual areas to 44 units recorded in three sessions from M1 in which the receptive location and size were not consistent with the overall topography of the offline receptive field map as to unambiguously assign the recording sites but included them when data were combined across areas.
Replication	The main results were reliably replicated in both animals as described in the manuscript and Supplementary Materials.
Randomization	All comparisons were made within subject across conditions, i.e. each subject was their own control.
Blinding	The experimenters were not blinded. Blinding was not relevant as no group allocation was done in our study

Reporting for specific materials, systems and methods

We require information from authors about some types of materials, experimental systems and methods used in many studies. Here, indicate whether each material, system or method listed is relevant to your study. If you are not sure if a list item applies to your research, read the appropriate section before selecting a response.

Materials & experimental systems

n/a	Involved in the study	
☒	☐	Antibodies
☒	☐	Eukaryotic cell lines
☒	☐	Palaeontology and archaeology
☐	☒	Animals and other organisms
☒	☐	Clinical data
☒	☐	Dual use research of concern

Methods

n/a	Involved in the study	
☒	☐	ChIP-seq
☒	☐	Flow cytometry
☒	☐	MRI-based neuroimaging

Animals and other research organisms

Policy information about studies involving animals; ARRIVE guidelines recommended for reporting animal research, and Sex and Gender in Research

Laboratory animals	Two adult rhesus monkeys (Macaca mulatta), 21 and 13 years old, weighing 9 kg each were used as subjects.
Wild animals	Study did not involve wild animals
Reporting on sex	Both animals used in the study were male. Sex of animals was not considered during study design as an effect of sex on low-level visual processing was not established.
Field-collected samples	Study did not involve samples collected in the field
Ethics oversight	All protocols were approved by the National Eye Institute Animal Care and Use Committee (animal M1) or by the relevant local authority (animal M2), the Regierungspräsidium Tübingen, Germany, and all experimental procedures were performed in compliance with the US Public Health Service Policy on humane care and use of laboratory animals

Note that full information on the approval of the study protocol must also be provided in the manuscript.
